# Impact of *Wuchereria bancrofti* Infection on Cervical Mucosal Immunity and Human Papillomavirus Prevalence in Women from Lindi and Mbeya Regions, Tanzania

**DOI:** 10.3390/tropicalmed10110317

**Published:** 2025-11-10

**Authors:** Maureen Mosoba, Thomas F. Marandu, Lucas Maganga, Jacklina Mhidze, Anifrid Mahenge, Jonathan Mnkai, Agatha Urio, Nhamo Chiwarengo, Liset Torres, Winfrida John, Abdallah Ngenya, Akili Kalinga, Upendo J. Mwingira, Manuel Ritter, Achim Hoerauf, Sacha Horn, Christof Geldmacher, Michael Hoelscher, Mkunde Chachage, Inge Kroidl

**Affiliations:** 1 Center for International Health, LMU University Hospital, 80802 Munich, Germany; gershomallianna@gmail.com (M.M.); hoelscher@lrz.uni-muenchen.de (M.H.); 2 Mbeya Medical Research Center, National Institute for Medical Research, Mbeya 53107, Tanzania; marandutf@udsm.ac.tz (T.F.M.); lmaganga@nimr-mmrc.org (L.M.); jmhidze@nimr-mmrc.org (J.M.); amahenge@nimr-mmrc.org (A.M.); jmnkai@nimr-mmrc.org (J.M.); aurio@nimr-mmrc.org (A.U.); nchiwerengo@nimr-mmrc.org (N.C.); 3 Mbeya College of Health and Allied Sciences, University of Dar es Salaam, Mbeya 53107, Tanzania; liset.torres4@gmail.com; 4 Mbeya Zonal Referral Hospital, Mbeya P.O. Box 419, Tanzania; 5 National Institute for Medical Research Headquarters, Dar es Salaam 11101, Tanzania; jwinrence@gmail.com (W.J.); abdallahngenya@gmail.com (A.N.); kalingaaka@yahoo.com (A.K.); 6 RTI International, Washington, DC 20005, USA; umwingira@yahoo.com; 7 Institute for Medical Microbiology, Immunology and Parasitology (IMMIP), University Hospital Bonn (UKB), 53127 Bonn, Germany; manuel.ritter@ukbonn.de (M.R.); achim.hoerauf@ukbonn.de (A.H.); 8 German-West African Centre for Global Health and Pandemic Prevention (G-WAC), Partner Site Bonn, 53127 Bonn, Germany; 9 German Center for Infection Research (DZIF), Partner Site Bonn-Cologne, 53127 Bonn, Germany; 10 Institute of Infectious Diseases and Tropical Medicine, LMU University Hospital, LMU Munich, 80802 Munich, Germany; horn.sacha@gmail.com (S.H.); geldmacher@lrz.uni-muenchen.de (C.G.); 11 German Center for Infection Research (DZIF), Partner Site Munich, 80802 Munich, Germany; 12 Immunology, Infection and Pandemic Research, Fraunhofer Institute for Translational Medicine and Pharmacology (ITMP), 80799 Munich, Germany; 13 Unit Global Health, Helmholtz Zentrum Munich, German Research Center for Environmental Health, 85764 Munich, Germany

**Keywords:** *Wuchereria bancrofti* infection, HIV, HPV, Immunomodulation, CCR5, Υδ T cells

## Abstract

We previously described an increased incidence of HIV among individuals infected with *Wuchereria bancrofti* (WB). However, no host, parasite, or viral factors were reported as directly associated with the increase in HIV incidence in this group. To investigate this, we compared T cell phenotypes between WB+ and WB− women. Flow cytometry analysis of activation and differentiation markers on CD4 T cells, as well as HIV entry receptor CCR5 was performed on cervical and peripheral blood samples from 54 women living without HIV (WLWoH). Additionally, HPV testing was performed on their specimens and for 13 WLWH. WB infection was associated with a significantly increased frequency of CD3^+^γδ2^+^ T cells in the cervical mucosa (median 4.0% vs. 1.4%, *p* = 0.012). Contrary to our expectations, we found lower frequencies of CCR5 on total, memory and activated memory CD4 T cells in the WB+ group. However, differences diminished after accounting for age and site of recruitment. WB and HIV infections were associated with an increased likelihood of high-risk human papillomavirus (HR HPV) positivity. (WB status: odds ratio (OR) 4.1, *p* = 0.066; HIV status: OR 5.5, *p* = 0.068). Our findings suggest immunological mechanisms by which WB increases the risk for other infections, e.g., HIV and HR HPV, albeit independent of the CCR5 receptor.

## 1. Introduction

Lymphatic filariasis (LF) is a chronic helminth infection transmitted by mosquitoes of the genera Anopheles, Culex and Aedes [1,2,3]. The infection is caused by the filarial nematodes *Wuchereria bancrofti* (WB) (accounts for 90% of infections), *Brugia malayi* and *Brugia timori* [4,5,6]. Globally, tremendous efforts have been undertaken to reduce the transmission of the filariae [7]. Kyela (situated in Mbeya region, southwest Tanzania) and Lindi (located in Lindi region, along the Indian Ocean, southeast Tanzania) were among the districts that were endemic for WB infection in Tanzania before treatment activities started [8,9]. After several rounds of anti-filarial drug distribution to all individuals in the districts, the prevalence dropped from 35.1% to 1.7% in Kyela district [10] and from 55.0% to 7.8% in Lindi district [11]. 

Helminths modulate the host immune response to prevent expulsion and enhance their survival [12,13,14]. This may potentially increase the host’s susceptibility to other infections such as malaria, tuberculosis and human immunodeficiency virus (HIV) [9,15,16,17]. Earlier reports of Ethiopian immigrants to Israel had illustrated that increased immune activation was responsible for the amplified susceptibility of helminth-infected individuals to HIV [18,19]. Later studies suggested that immune modulation engendered by helminths creates a favourable milieu for productive HIV infection [20,21]. This theory was supported by a prospective cohort study conducted by our group, which revealed a 2.3-fold increase in the incidence of HIV in WB-infected compared to uninfected individuals [9]. After the elimination of the filarial infection in this area, the HIV incidence in the group of previously infected persons decreased, which is supportive of the initial findings [22]. 

Two receptors, CCR5 and α4β7, expressed on CD4 T cells are associated with increased susceptibility to HIV infection [23]. HIV requires chemokine receptor CCR5 to gain entry into CD4 T cells [24]. Kalinkovich et al. demonstrated that helminth infection augments the expression of CCR5, thereby increasing vulnerability to HIV infection [19]. Several publications have focused on the effect of helminths on the systemic immune response in the peripheral blood but not in the cervical mucosa [20,25,26,27,28,29,30].

Significantly higher levels of immune activation were seen in WB-infected individuals compared to uninfected individuals in a study from 2009 [28]. It is important to note that the participants from this previous study had been recruited during a time of high WB prevalence and worm burden in single individuals in Kyela district. At the time, the prevalence of WB was 35.1% among individuals aged 14–65 years, of which the majority were highly positive for circulating filarial antigen [10]. A decade later, following seven rounds of antifilarial treatment and three years of transmission assessment survey, WB prevalence plummeted to 1.7% [10]. However, comparing the immune milieu of the peripheral blood and local mucosa reveals important differences regarding distribution of different cell types, receptors, activation and exhaustion markers [31,32,33]. For addressing the susceptibility towards sexually transmitted infections (STIs), cervical cell collections are of more relevance than measurements in the peripheral blood [32]. Lately, studies have described changes in the cervical mucosal immunity of individuals infected with *Schistosoma haematobium*, *Schistosoma mansoni,* and hookworms [34,35]. However, no study has evaluated the impact of WB infection on the immune response in the female reproductive tract (FRT).

The FRT is known as the main portal of entry for STIs, including HIV and human papillomavirus (HPV) in women, and its mucosa provides a protective barrier against sexually transmitted pathogens [36,37,38]; therefore, any breach in its integrity, such as a disruption in the epithelium or genital inflammation, could lead to a heightened risk for HIV and HPV infection [39,40,41]. Anatomically, the FRT consists of the upper (endocervix and uterus) and lower (ectocervix and vagina) tracts, which are histologically and immunologically distinct [42]. Mononuclear cells are variably distributed in the upper and lower tracts and include αβ and γδT cells and B cells. CD4^+^ and CD8^+^ T lymphocytes are central in controlling and clearing infections in the FRT [43,44], whereas γδ T cells, which are resident in the intraepithelial layer of the FRT mucosa [45,46,47], provide a defensive barrier and exert a potent cytolytic effect against pathogenic microorganisms [48].

It has also been postulated that immune modulation by helminths results in changes in the FRT immune milieu, creating a favourable environment not only for HPV infection but also for its persistence [34,49,50]. Persistent HPV infection, especially with the HR HPV genotypes, is the most important risk factor for cervical cancer [51]. Cervical cancer ranks eighth among neoplasms in women globally and is the leading cause of cancer-related deaths in Tanzanian women [52]. In 2020, the prevalence of HPV infection in women in Tanzania (20–65 years of age) was 18.9%, with HPV 52 (3.8%), HPV16 (3.6%), HPV 58 (2.5%) and HPV18 (2.4%) being the most common genotypes in the FRT of women without precancerous lesions [53]. The association between WB and HPV infections has never been explored before. 

Thus, this study evaluated the immune profile in the FRT in WB infection and investigated whether WB infection potentially results in alterations in the immune milieu of the FRT, which might consequently increase the predisposition to sexually transmitted infections, particularly HIV and HR HPV. Herein, we compared the expression of markers for T cell activation, T cell differentiation (CD4, CD8 T and γδT cells) and HIV coreceptor CCR5 and facilitator, integrin α4β7. These markers were evaluated in the cervical mucosa and peripheral blood between WB-infected and uninfected women.

## 2. Materials and Methods

### 2.1. Study Volunteers and Baseline Tests

This sub-study included female participants aged 18 years and above from Kyela and Lindi districts in Tanzania who were part of a study (Risk of HIV Infections through Nematode Organism (RHINO)) that enrolled volunteers based on their HIV and WB infection status, as previously described [10]). HIV status was determined following the Tanzanian national HIV testing algorithm. Participants were first tested using HIV—1/2 3.0 (Standard Diagnostics Inc., Bioline^TM^, Taunton, MA, USA), and positive results were confirmed by Uni-Gold^TM^ Recombigen^R^ HIV—½ (Trinity Biotech, Inc., New York, NY, USA). Any discrepancies between the two tests were resolved by Western Blot (MPD HIV Blot 2.2, MP Biomedicals, Irvine, CA, USA). Filariasis Test Strip (FTS, Abbott Laboratories, Chicago, IL, USA) was used for the detection of circulating filarial antigen to determine WB infection status. Participants who tested positive were referred to as WB-infected (WB+), while those who tested negative were referred to as WB-uninfected (WB−). Beta human chorionic gonadotropin pregnancy detection urine test (Viola^R^—C, Moellersdorf, Austria) was used to rule out pregnancy. Participants with a fever—defined as a temperature above 37 °C—were excluded. Malaria was ruled out using Malaria Pf Ag Rapid Antigen test (City Bioline^TM^, Taunton, MA, USA).

### 2.2. Sample Collection

Next, 20 mL of venous blood was collected into sodium heparin vacutainers (Sarstedt, Nürnberg, Germany), of which 4 mL was aliquoted for T cell immunophenotyping and 16 mL for peripheral blood mononuclear cell isolation. Papanicolaou smear was performed by well-trained study clinicians by collecting ecto- and endocervical cells using an Ayres spatula. Cervical cells were then applied to a glass slide and fixed in 70% alcohol in a 50 mL Falcon tube. Thereafter, endocervical cells were collected by inserting a cytobrush into the cervical os gently rotating through 360°, whereby part of the specimen was used for Papanicolaou testing while the remaining cells were collected for immunophenotyping in 5 mL complete media (10% heat-inactivated foetal bovine serum (Sigma, Aldrich, Munich, Germany) in RPMI- 1640 medium (Gibco, Invitrogen, Carlsbad, CA, USA), 50 U/mL Penicillin, 50 μg/mL Streptomycin and 1× antibiotic- antimycotic solution (Sigma, Aldrich, Munich, Germany). A second cytobrush sample was collected for HPV genotyping and stored in 5 mL PreservCyt cell collection media (Roche). Samples were transported to the laboratory at room temperature (blood specimens, Pap smear and cytobrush for HPV genotyping) or 2–4 °C (cytobrush specimens for immunophenotyping) within 6 h of collection. 

### 2.3. Flow Cytometry

Peripheral blood for flow cytometry was processed and stained according to Horn et al. 2021 [26], while cervical mucosal cells collected were processed and stained according to Mbuya et al. [33]. Fluorochrome labelled antibody panels used for staining endocervical cytobrush and peripheral blood samples were as follows: CD3 ECD (Beckman Coulter, UCHT1, Marseille, France), CD4 Per CP C5.5 (eBioscience, OKT4, Carlsbad, CA, USA), CD45RA PB450 (BD Biosciences, HI100, San Diego, CA, USA), HLA-DR PeCy7 (eBioscience, LN3, San Diego, CA, USA), integrin B7 PE (Biolegend, FIB27, San Diego, CA, USA), CCR5 APC (Miltenyi Biotec, REA245, Bergisch Gladbach, Germany), CD27 APC H7 (BD Pharmingen^TM^, M-T271, San Jose, CA, USA). Forkhead box P3 (FoxP3) Alexa Fluor 488 (Biolegend, 259D, San Diego, CA, USA) and CD25 BV605 (BD Biosciences, BC96, San Diego, CA, USA) were only added to peripheral blood samples. γδ1—FITC (Invitrogen, TS8.2, Chicago, IL, USA), γδ2—Alexa Fluor 700 (Biolegend, B6, San Diego, CA, USA) and LIVE/DEAD^R^ fixable aqua dead cell stain (ThermoFischer Scientific Inc., Eugene, OR, USA) were only added to cervical cytobrush samples. Acquisition of cells was performed using a 13-colour CytoFLEX (A00-11102, Beckman Coulter, Inc., Suzhou Xitogen Biotechnologies Co., Ltd., Suzhou, China) flow cytometer. Compensation was conducted with antibody capture beads (Beckman Coulter Inc., Brea, CA, USA), where staining was performed separately with the individual antibodies. FlowJo software version 10.8.1 (Becton, Dickinson and Company, Ashland, OR, USA) and Kaluza software version 2.1 (Beckman Coulter Inc., Brea, CA, USA) were used to analyse flow cytometry data. 

### 2.4. Human Papillomavirus DNA Detection and Genotyping

DNA extraction and purification from endocervical cells were performed using QIAamp DNA mini kit and thereafter stored at −20 °C in elution buffer (Qiagen, Hilden, Germany). Quantitative polymerase chain reaction assay with Seegene Anyplex™ II HPV HR Detection (Seegene Inc., Seoul, Republic of Korea) was then carried out to detect the following 14 HR HPV genotypes—16, 18, 31, 33, 35, 39, 45, 51, 52, 56, 58, 59, 66 and 68—as described by Chachage et al [54]. 

### 2.5. Histopathology of Papanicolaou Smear

Histology of Papanicolaou (Pap) smears based on haematoxylin and eosin staining was performed in the Pathology Department of the Mbeya Zonal Referral Hospital. The Bethesda system for reporting cervical cytology was used following evaluation (Pangarkar MA, 2022) [55].

### 2.6. Statistical Analysis

GraphPad Prism version 8.4.2 (GraphPad Software, Inc., La Jolla, CA, USA) was used to compare single parameters between groups. Analysis of unpaired measurements was performed by Mann–Whitney U-test. Fisher’s exact and Chi square test were used to determine associations between categorical variables. A test of proportions was performed to assess whether the study cohort was representative of the population. STATA software version 17.0 (StataCorp LP, College Station, TX, USA) was used to perform multivariable linear regression for measuring the association between WB infection and continuous variables. Firth’s logistic regression was used for analysing the association between WB infection and categorical variables. *p*-value ≤ 0.05 was considered statistically significant.

## 3. Results

### 3.1. Study Cohort

**Figure 1:**
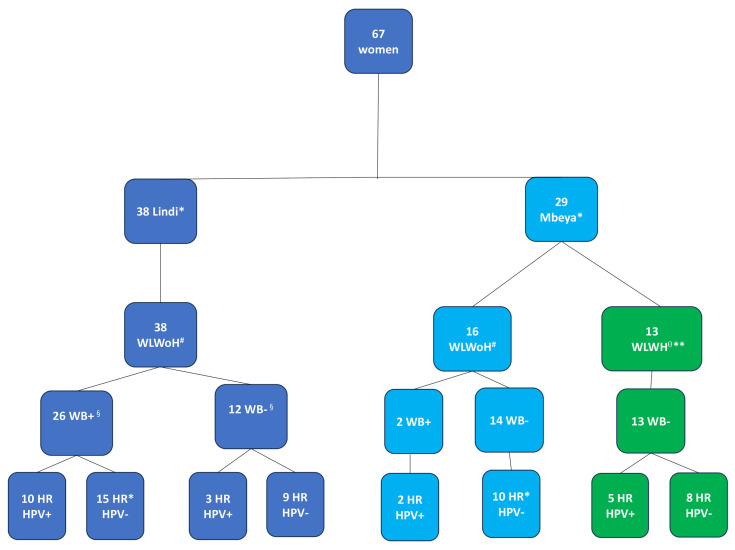
Participant groups with respect to WB infection status (WB+/WB−), site of recruitment and HR HPV infection status (HR HPV detected {HR HPV+}/HR HPV not detected {HR HPV−}). Data analysis was performed on women with flow cytometry parameters. Mucosal sample collection was not possible for five of the women because they were on their menstrual period at the time of sample collection. * For one WB+ woman from Lindi and 4 WB− women from Mbeya, the HPV testing was not performed; ^#^ WLWoH = Women living without HIV; ^θ^ WLWH = Women living with HIV. ** WLWH were tested for HPV but excluded from the immunological analysis. ^§^ For three samples, the CD4 clone did not bind, and immunological analysis was not possible.

An investigation of immune parameters was carried out for a subgroup of 29 female participants from the main RHINO-study cohort, which comprised 1299 individuals from Kyela, previously described by Mnkai et al. [10].and an additional 38 women from the partner study conducted in Lindi, Tanzania (Figure 1). We solely focused on female participants with available cervical mucosa samples to address cervical mucosal immunity. Thus, our sub-study was composed of 67 women, 13 of whom were HIV-positive and were excluded from the analysis of immune parameters. However, because they underwent screening for cervical cancer and HPV, their Pap smear and HPV results were included (Figure 1). 

For 54 HIV negative women aged between 18 and 65, a blood sample was collected. Peripheral blood immunophenotyping was performed in 51 of these women; for 3 participants, the CD4 antibody clone did not bind; hence, the dependent measurements were not used (Figure 1). Twenty-six (51.0%) were WB-infected (WB+) with a median age of 34.5 (IQR: 29–44) years compared to 25 WB-WB-uninfected (WB−) women, whose median age was 32.0 (IQR: 23–41) years, *p* = 0.309. 

Cervical immunology was analysed in 40 of the 54 HIV negative women for maturation markers and HIV receptor and facilitator. γδ1 and γδ2 receptors were measured in 35 women. Cervical cytobrush samples were not collected in four women because they were on their menstrual period. During processing of the cervical cytobrush samples, 10 samples were excluded: 5 samples were contaminated with peripheral blood, and 5 samples had few cells. WB+ women (*n* = 23) had a median age of 34.0 (IQR: 29–44) years compared to WB− women (*n* = 17), having a median age of 32.0 (IQR: 23–39) years, *p* = 0.193. In a separate analysis, 62 women from our cohort were screened for HR HPV and cervical carcinoma. In this category, 49 were women living without HIV (WLWoH), while 13 were women living with HIV (WLWH).

### 3.2. Impact of WB on the Maturation Status of CD4 T Cells in the Cervical Mucosa and Peripheral Blood

**Figure 2:**
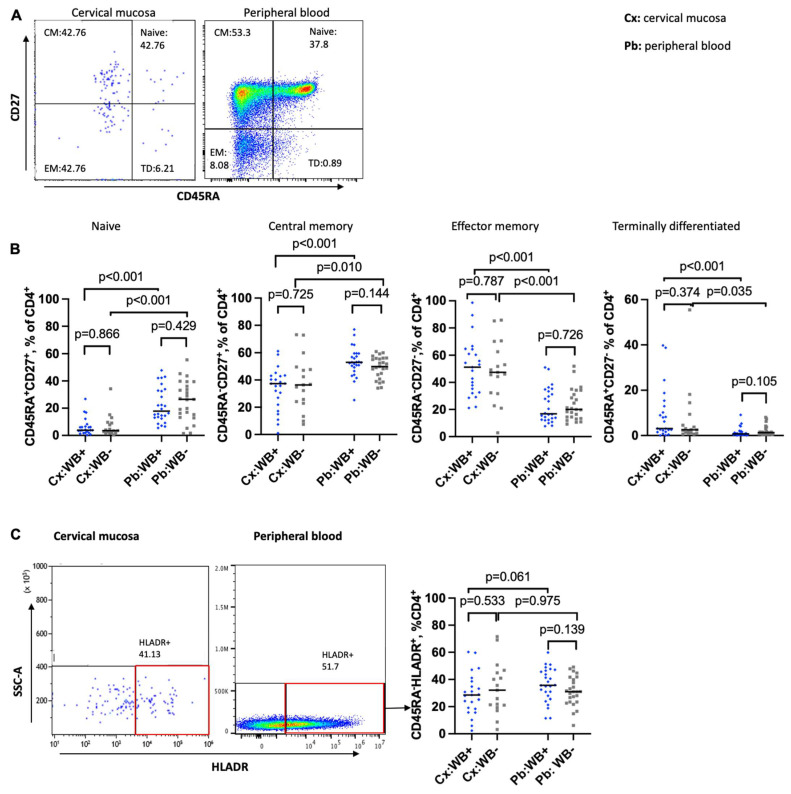
CD4 T cell differentiation and activation in the cervical mucosal and peripheral blood. (**A**): Representative flow plot displaying the distribution of naïve (Q2), central memory (CM) (Q1), effector memory (EM) (Q4), and terminally differentiated (TD) (Q3) CD4 T cells. (**B**): Comparison of frequencies of naïve, central memory (CM), effector memory (EM), and terminally differentiated (TD) CD4^+^ T cells in the cervical mucosa (Cx) and peripheral blood (Pb) between WB+ and WB− groups. (**C**): Representative flow plots and a graph showing frequencies of activated memory CD4 T cells (CD4^+^CD45RA^−^HLA-DR^+^) in the cervical mucosa and peripheral blood. Cx:WB+ *n* = 23, Cx:WB− *n* = 17, Pb:WB+ *n* = 26, and Pb:WB− *n* = 25. Each grey square and blue diamond represents a participant, and a horizontal line is the group median. Groups were compared using Mann–Whitney U-test.

CD45RA was used for differentiation of naïve and memory cells, with CD4^+^ CD45RA^−^ T cells defined as “memory” CD4 T cells. To determine the influence of WB infection on more distinct CD4 T cell subsets, naïve, central memory (CM), effector memory (EM), and terminally differentiated (TD) CD4 T cells were discriminated based on their expression of CD45RA and CD27 in the cervical mucosa and peripheral blood (Figure 2A). The subsets were defined as follows: naïve (CD45RA^+^CD27^+^), CM (CD45RA^−^CD27^+^), EM (CD45RA^−^CD27^−^), and TD (CD45RA^+^CD27^−^) (Figure 2A). 

In the initial analysis, we saw comparable frequencies of naïve CD4, CM, EM, and TD T cells in WB+ and WB− women (Figure 2B) for both compartments. Among the youngest group of women between 18 and 25 years, the unadjusted analysis suggested an impact of WB infection (Figure S1). However, since the differentiation of cells changes with age, we performed logistic regression analysis adjusting for that influence [56]. In the peripheral blood, we found significantly reduced frequencies of naïve CD4 cells (*p* = 0.033) and increased frequencies of EM cells (*p* = 0.021) among the women aged 45 to 65 years (compared to the younger age groups). However, filarial infection did not have a significant impact on any of the CD4 T cell types. 

Comparing the cells of the cervix with the peripheral blood cells, we noted significant differences between the two compartments, whereby the frequencies of naïve and CM were lower in the cervical mucosa (naïve: 3.9% vs. 17.9%, *p* < 0.0001; CM: 37.1% vs. 53.0%, *p* < 0.0001;) but frequencies of EM and TD were higher in the cervical mucosa (EM: 51.2% vs. 16.8%, *p* < 0.0001; TD: 3.1% vs. 0.7%, *p* < 0.0001, Figure 2B).

Upon analysis of the frequencies of activated memory CD4 T cells (CD4^+^CD45RA^−^HLA-DR^+^), we did not detect a significant difference between WB+ and WB− groups in both compartments: (cervix (Cx): median 28.5% vs. 32.1%, *p* = 0.533; peripheral blood (Pb): median, 35.7% vs. 30.9%, *p* = 0.139) (Figure 2C). This was still true after adjusting for age and recruitment site (cervical mucosa (Cx), *p* = 0.956; peripheral blood (Pb), *p* = 0.105). We noted a trend of fewer activated memory cells among the age group 45 to 65 years.

### 3.3. Influence of WB Infection Status on CD4 T Cells Expression of CCR5 and a4b7

**Figure 3:**
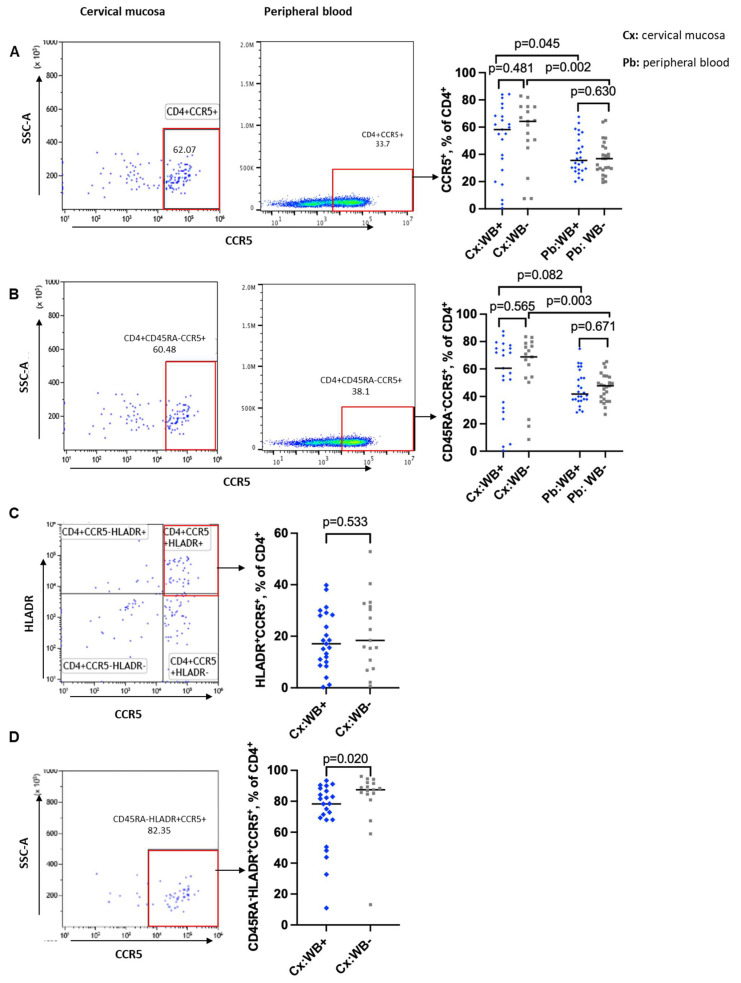
Frequencies of CCR5^+^ CD4 T cells in cervical mucosal and peripheral blood in relation to WB infection status. Representative flow plots and graphs showing frequencies of CCR5-expressing CD4 T cells in total CD4 T cells (**A**), memory CD4 T cells (**B**) from the cervical mucosa and peripheral blood of WB+ and WB− participants. (**C**): Representative flow plot and a graph comparing frequencies of CD4 T cells co-expressing activation marker (HLA-DR and CCR5) between WB+ and WB− groups in the cervical mucosa. (**D**): Representative flow plot and a graph comparing frequencies of memory-activated CD4 T cells expressing CCR5 between WB+ and WB− groups in the cervical mucosa. Cx:WB+ *n* = 23, Cx:WB− *n* = 17, Pb: WB+ *n* = 26, and Pb:WB− *n* = 25. Each grey square and blue diamond represent a participant and a horizontal line is the group median. Groups were compared using Mann–Whitney U-test.

We used multiparametric flow cytometry to analyse the frequencies and mean fluorescence intensity (MFIs) of CCR5 on total (CD4^+^CCR5^+^), memory (CD4^+^CD45RA^−^CCR5^+^), activated (CD4^+^CCR5^+^HLA-DR^+^) and activated memory (CD4^+^CD45RA^−^HLA-DR^+^CCR5^+^) CD4 T cells (gating strategies for CD4 T cells are shown in Figure S2). The frequencies of CCR5 on total and memory CD4 T cells were measured in both compartments. Results were comparable between WB+ and WB− groups, however with a trend towards more CCR5-positive cells among the WB− subgroup in the cervical mucosa: Total CD4 T cells (Cx: median 58.2% vs. 64.3%, *p* = 0.481; Pb: median 35.5% vs. 36.9%, *p* = 0.630) (Figure 3A) and memory CD4 T cells (Cx: 60.5% vs. 68.8%, *p* = 0.565; Pb: 41.8% vs. 47.8%, *p* = 0.671) (Figure 3B). Regression analysis revealed that the trends toward lower frequencies of CC5 positive cells among the filarial uninfected women were diminished when adjusted for age and recruitment site, as shown in Table 1.

The binding of the HLA-DR antibody was problematic in one of the panels of the peripheral blood. For that reason, we can present the measurements of CCR5 on activated and activated memory CD4 T cells only for the mucosal compartment (Figure 3C,D). No difference between WB+ and WB− groups was seen among activated CD4 T cells (Cx: median 19.1% vs. 18.0%, *p* = 0.533, Figure 3C). Interestingly, we found significantly lower frequencies of CCR5 on activated memory CD4 T cells in WB+ compared to WB− women in the cervical mucosa using Wilcoxon rank sum test (Cx: median 78.3% vs. 87.5%, *p* = 0.020) (Figure 3D). Including age and recruitment site into the regression analysis, the above-mentioned difference reduced to a trend (Table 1). A significant impact of age on all our measurements of CCR5 on different cell types could be demonstrated (Figure S3). Comparison of the MFI of CCR5+ in the total CD4 T cells (Figure S4A) and in the memory CD4 T cells (Figure S4B) did not reveal any difference between filarial-infected and uninfected subgroups.

When comparing the two compartments, the cervical mucosa had significantly higher frequencies of CCR5 expression on the different CD4 T cell populations compared to the peripheral blood. We found a significant difference on total CD4 T cells (WB+: *p* = 0.045; WB−: *p* = 0.002, Figure 3A) and on memory CD4 T cells (WB+: *p* = 0.082; WB−: *p* = 0.003, Figure 3B). 

A similar analysis was carried out for the HIV facilitator α4β7 (Figures S4 and S5). Frequencies of α4β7 on total (Cx: median 17.9% vs. 14.1%, *p* = 0.787; Pb: median 24.3% vs. 21.8%, *p* = 0.604), memory (Cx: median 15.3 vs. 14.3%, *p* = 0.746; Pb: median 19.1% vs. 19.2%, *p* = 0.929 (Figure S6A) and activated memory (Cx: median 17.3% vs. 26.4%, *p* = 0.265; Pb: median 15.2% vs. 17.5%, *p* = 0.247, Figure S6B) CD4 T cells were comparable between WB+ and WB− groups in both compartments. 

### 3.4. Infection with WB Increases the Frequency of γδ2 CD3 T Cells in the Cervical Mucosa

**Figure 4:**
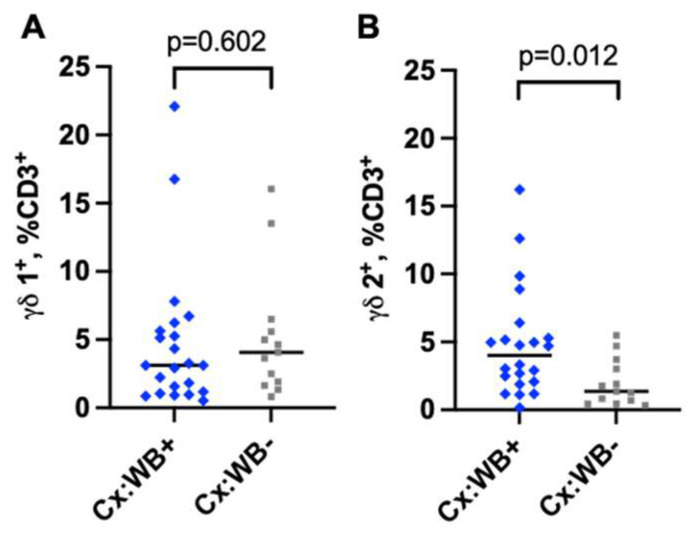
Frequency of γδ T cells in the cervical mucosa. Frequencies of γδ1 (**A**) and γδ2 (**B**) T cells from the cervical mucosa of WB+ (*n* = 22) and WB− (*n* = 13) women. Each grey square and blue diamond represents a participant and a horizontal line is the group median. Groups were compared using Mann–Whitney U-test.

We next evaluated the proportion of γδ1^+^ and γδ2^+^ CD3 T cells in WB infection in the cervical mucosa (gating strategy in Figure S7). The frequency of CD3^+^γδ1^+^ T cells was comparable between the groups (median 3.1% vs. 4.0%, *p* = 0.602, Figure 4A). However, the frequency of CD3^+^γδ2 was significantly elevated in the WB+ group (median 4.0% vs. 1.4%, *p* = 0.012, Figure 4B). A multivariable linear regression analysis (to adjust for age and recruitment site) confirmed that WB infection was associated with a significant increase in the frequency of CD3^+^γδ2^+^ T cells, *p* = 0.022, Table 2).

**Figure 5:**
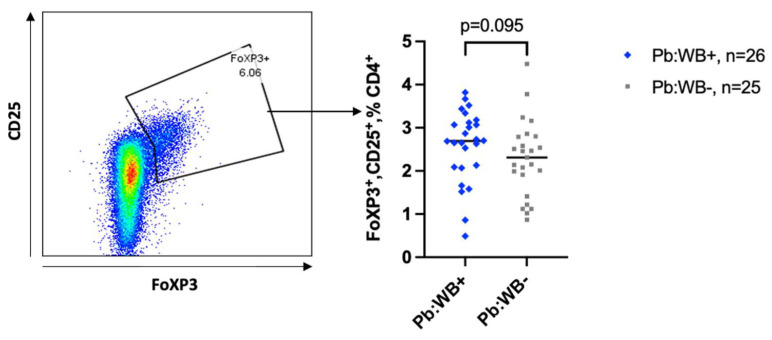
Frequencies of regulatory T cells in the peripheral blood. Representative flow plot showing the fraction of T regs and the corresponding graph comparing the frequency of T regs between WB+ and WB− groups in the peripheral blood. Each grey square and blue diamond represents a participant, and the horizontal line is the group median. Groups were compared using Mann–Whitney U-test.

Regulatory CD4 T cells (defined by their expression of transcription factor FoxP3 and CD25) were only analysed in the peripheral blood. We observed a non-significant trend towards an increase in the frequency of regulatory CD4 T cells (T regs) in the WB+ women (Figure 5), which did not change after adjusting for age and recruitment site.

### 3.5. Increased HR HPV Prevalence in WB-Infected Women

**Figure 6:**
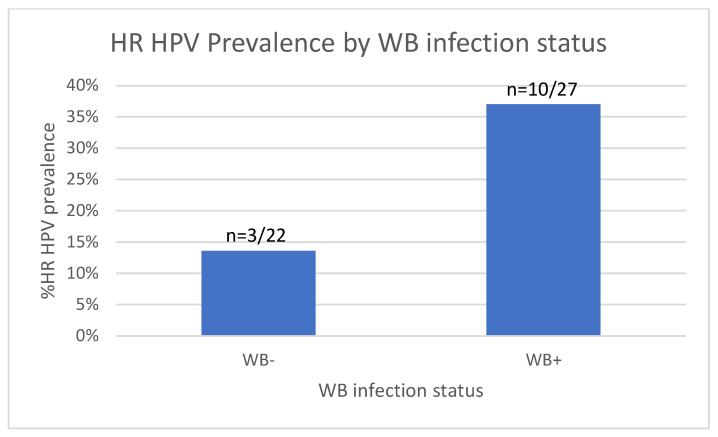
HR HPV status in the WB+ and WB− groups. Bar graph showing the number of WB+ and WB− women testing positive for HR HPV among WLWoH.

We next analysed the prevalence of HR HPV infection between the WB+ and WB− groups. On assessment of HR HPV prevalence among the 49 WLWoH, a trend towards increased prevalence of HR HPV was observed in the WB+ group, whereby 37.0% (10/27) tested positive for HR HPV compared to 13.6% (3/22) in the WB− group (*p* = 0.104, Fisher’s exact test, Figure 6).

Addressing the impact of HIV on HPV prevalence, we noted that 5 HR HPV infections (38.5%) were among the 13 WLWH compared to the 13/49 (26.5%) HR HPV positive among the WLWoH (*p* = 0.400, chi2).

Taking into account age, WB infection, and HIV status, Firth’s logistic regression model was used to demonstrate an increased probability of HP HPV positivity in individuals with WB infection (odds ratio 4.1, *p* = 0.066, Table 3) and HIV infection (odds ratio 5.5 *p* = 0.068, Table 3). However, neither factor reached statistical significance.

HR HPV genotypes detected in our cohort were: HPV 52, 68, 16, 18, 45, 35 and 58, 31, 56 and 39. HPV 52 was the most frequent genotype, accounting for 23.1% (3/13) of those that tested positive followed by HPV 68, which accounted for 15.4% (2/13). HPV genotypes 16, 18, 35, 56, and 58 accounted for 7.7% (1/13) of the HR HPV infections each. Two participants were double-infected with 2 HR HPV genotypes (52, 31 and 56, 39), which also accounted for 7.7% (1/13) of the HR HPV infections each. Pap smears were performed on 49 women and 45 of these had normal cytology, while four smears were unsatisfactory for evaluation.

## 4. Discussion

Following the description of the increased incidence of HIV in people infected with filariae in Tanzania [9], we attempted to determine the immunological mechanism behind this epidemiological finding. While we found little to no influence of WB status on the expression of HIV facilitators, WB infection was associated with increased frequencies of CD3^+^γδ2^+^ T cells in the cervical mucosa and an increased risk of HR HPV, indicating a possible mechanism by which WB may not only increase HIV risk but also that of other STIs such as HPV.

HIV requires additional structures to enter the CD4 cell. CCR5 is a chemokine receptor that serves as a coreceptor for HIV and facilitates viral entry into the cell [57,58]. Blocking CCR5 is used therapeutically as an antiretroviral strategy [59]. The α4β7 integrin is a heterodimeric glycoprotein found on activated CD4 T cells, which also express high levels of CCR5 [23], and acts as a homing receptor that mediates lymphocyte migration. We therefore focused on measuring these two markers on T cells of the cervical mucosa and peripheral blood. We have already published that naïve CD4 T cells hardly express CCR5, whereas central memory CD4 T cells contain a significant proportion of CCR5^+^ cells [20]. Furthermore, it is known that low systemic immune activation correlates with protection against HIV [60,61,62] and that individuals with chronic immune activation have a higher risk of acquiring HIV [63]. We therefore compared the degree of immune activation, the differentiation of CD4 T cells and other T cell types between WB-infected and uninfected women. 

Contrary to our expectations, we did not find increased frequencies of CCR5^+^ cells in any of the cell subtypes. If anything, frequencies were lower in women infected with filariae. However, these trends were attenuated when age and site of recruitment were taken into account. It is worth noting that the cervical mucosa had remarkably higher CCR5 frequencies on total and memory CD4 T cells than peripheral blood, as was previously reported by Mbuya et al. [33]. This underlines the importance of examining immune cells in the “right” compartment where the transmission of a pathogen takes place.

An interesting finding was the higher prevalence of HR HPV infections in women infected with filariae. Induction of systemic changes in the immune milieu of the FRT mucosa by helminths has been shown to influence susceptibility to other pathogens [64]. Chronic infection with hookworms has been associated with an increased prevalence of HR HPV and a higher viral load of HPV16 and 18 [34,49,50]. Interestingly, our study showed similar results for infection with WB, with the prevalence of HR HPV being significantly higher in WB-infected women than in uninfected women. In a multivariable regression analysis adjusting for age, WB and HIV status, WB and HIV infections were both associated with an increased likelihood of HR HPV infection.

The influence of filarial infection, not only on HIV but also on HPV susceptibility, leads to a different view of the above results. Since CCR5 is not responsible for the increased acquisition of pathogens other than HIV, it can be ruled out that CCR5 is the responsible factor for our epidemiological findings. 

One of the most important determinants of HIV infection is the activation status of a cell, as the acquisition and transmission of HIV is enhanced in activated CD4 T cells [23,32,65,66,67]. In areas where HIV and filarial infections co-occur, previous studies have found an expansion of activated CD4 T cells (as measured by HLA-DR and HLA-DR/CD38 expression) in WB infections [28]. However, in this current study, we did not find a difference in CD4 T cell activation in the WB+ group. This difference from the results of our original hypothesis could be due to a lower worm burden in the population, even in helminth-infected individuals, approximately 10 to 16 years after starting MDA with ivermectin and albendazole. Therefore, it is likely that some of the immune mechanisms triggered by the helminths were attenuated [68,69].

Helminth infections, in general, have been associated with an expansion of Tregs. A relationship between filariasis and T reg expansion was shown by Babu et al. and Metenou et al. [70,71]. Herein, we did see a trend towards increased frequencies of T regs in WB infection. 

Our main immunological finding is that WB infection is associated with a remarkable expansion of CD3^+^ γδ2 T cells in the FRT mucosa. CD3^+^ γδ T cells play a crucial part in the immune response against helminth, protozoan, bacterial and viral infections. Previous studies have described the role of CD3^+^ γδ T cells in infections elicited by different pathogens in murine models; Ferrick et al. reported that CD3^+^ γδ T cells promote the production of IFN-γ and IL-4 in response to *Listeria monocytogenes* and *Nippostrongylus brasiliensis,* respectively [72]. Sciammas et al. demonstrate that CD3^+^ γδ T cells mitigate the replication of herpes simplex virus 1 (HSV-1) infection and inhibit HSV-1-induced encephalitis [73]. Lockhart et al. found that CD3^+^ γδ T cells curtail the progression of *Mycobacterium tuberculosis* infection and were responsible for the production of proinflammatory cytokines [74]. In malaria-infected human subjects, CD3^+^ γδ T cells were increased 3-fold and were involved in limiting the infection through phagocytosis of Plasmodium-infected red blood cells [75]. These aforementioned studies analysed CD3^+^γδ T cells in the peripheral blood. To our knowledge, this is the first study to investigate CD3^+^ γδ T cells in the FRT mucosa in WB infection. We found a significantly higher frequency of CD3^+^ γδ2^+^ T cells in the FRT of WB-infected women, which was supported by multivariable regression analysis that adjusted for age and recruitment site (Table 2). According to recent studies in human and murine models, CD3^+^ γδ T cells are principally found in mucosal tissue sites, but only a small fraction circulates in peripheral blood [76,77]. Augmentation of CD3^+^ γδ2^+^ T cells might be a result of their infiltration from the periphery to the FRT mucosa in response to the filarial worm infection, consequently promoting inflammation in the mucosal site; this could in turn facilitate acquisition of HIV in the preferentially targeted cells [63,78]. Previous work has shown that γδ2^+^ T cells are a potential target for HIV [79]. Thus, an increase in the frequency of γδ2^+^ T cells might heighten the risk of HIV infection and transmission. By strengthening measures towards eliminating WB infection, the incidence of HIV will consequently be reduced.

Our study was subject to several limitations. The main limitation was the low prevalence of WB infection in the primary study area (Kyela) [10], which necessitated recruiting participants for our study from an area where WB was still moderately endemic (Lindi) [11]. Based on prevalence data of WB in Kyela district from our previous studies, we assumed that there would be more women infected with WB in this area. A surveillance conducted in 2009 had shown a WB prevalence of 35.1% in the population of Kyela over the age of 14 years [10]. However, the MDA was more effective than we had originally assumed. Among individuals aged 14–65 years, WB prevalence decreased from 35.1 to 1.7% in that area [10], resulting in a small number of filaria-positive women in our primary study area when recruitment began in 2019. Participants from a partner study focusing on the remaining “hot-spots” of WB infection in the coastal areas of Tanzania were invited [11]. Unfortunately, the outbreak of SARS-CoV-2 led to travel restrictions, which meant that fewer women were willing to travel several days across the country to undergo gynaecological tests. All of this resulted in a small sample size. When planning the study, we had attempted to estimate the required sample size. Since the effects of WB infection on HR HPV prevalence had not been studied previously, these calculations were difficult to perform. However, we aimed for a sample size of more than 70 women, as these numbers had been calculated to measure the effects of HIV with significant results. 

We compared the effect of WB infection between the peripheral blood and cervical mucosa, which has not been carried out before. Furthermore, we demonstrate the impact of age on T cell differentiation, as seen in other studies. We noticed remarkable differences between certain T cell subsets between the cervical mucosa and peripheral blood, which corroborates previous findings.

In conclusion, our data demonstrate that WB infection might be linked to an increase in the expression of CD3^+^ γδ2^+^ T cells, suggesting a possible immunological mechanism through which WB infection enhances the risk for HIV infection. Furthermore, WB and HIV infections independently showed a trend towards an increase in the prevalence of HR HPV infection. 

## Figures and Tables

**Table 1:** Uni- and multi-variable linear regression results for the association of *W. bancrofti* infection and frequency of CCR5-positive cells among various CD4 T cell subgroups in the genital mucosa adjusted for age and recruitment site.

			Univariable		Multivariable	
Covariate	N = 40	Mean	Coefficient (95% CI)	*p* Value	Coefficient (95% CI)	*p* Value
Cervix cells						
CD4+CCR5+						
WB negative *	17	56.3	Ref		Ref	
WB positive	23	50.3	−6.0 (−22.3–10.4)	0.464	0.2 (−16–16.5)	0.977
CD4+CD45RA-CCR5+						
WB negative *	17	60.5	Ref		Ref	
WB positive	23	54.3	−6.2 (−22.8–10.4)	0.456	1.8 (−14.4–17.9)	0.827
CD4+CCR5+HLA-DR+						
WB negative *	17	21.4	Ref		Ref	
WB positive	23	18.3	−3.1 (−11.2–5.1)	0.452	−1.8 (−11–7.3)	0.686
CD4+CD45RA-HLA-DR+CCR5+						
WB negative *	17	81.5	Ref		Ref	
WB positive	23	71.2	−10.3 (−23.6–3.0)	0.127	−8.7 (−23.2–5.7)	0.227

N = number of women, CI = confidence interval, * Reference strata.

**Table 2:** Uni- and multi-variable linear regression results for the association of *W. bancrofti* infection and frequency of CD3+ γδ2 T cells in the genital mucosa adjusted for age and recruitment site.

			Univariable		Multivariable	
Covariate	N = 35	Mean	Coefficient (95% CI)	*p* Value	Coefficient (95% CI)	*p* Value
WB						
Negative *	13	2.0	Ref		Ref	
positive	22	4.8	**2.8 (0.4–5.2)**	**0.022**	**3.0 (0.5–5.6)**	**0.022**
Age-group						
18–<25 *	9	3.5	Ref		Ref	
25–<45	19	4.6	1.1 (−1.8–4.0)	0.452	−0.2 (−3.1–2.7)	0.909
45–65	7	1.8	−1.8 (−5.4–1.8)	0.328	−2.9 (−6.4–0.6)	0.104
Site						
Kyela *	3	2.3	Ref		Ref	
Lindi	32	3.9	1.7 (−2.7–6.1)	0.449	0.3 (−3.9–4.5)	0.885

N = number of women, CI = confidence interval, * Reference strata.

**Table 3:** Uni- and multi-variable Firth’s logistic regression for the association of age, *W. bancrofti* and HIV infection status with HR HPV prevalence.

			Univariable		Multivariable	
Covariate	N = 61 *	N-positive (%)	Odds Ratio (95% CI)	*p* Value	Odds Ratio (95% CI)	*p* Value
WB						
Negative *	34	8 (23.5)	Ref		Ref	
positive	27	10 (37.0)	1.87 (0.63–5.55)	0.259	4.07 (0.91–18.1)	0.066
Not performed **	1 *	-	-	-	-	**-**
HIV status						
Negative *	49	13 (26.5)	Ref		Ref	
positive	12	5 (41.7)	1.98 (0.56–7.03)	0.289	5.46 (0.88–33.66)	0.068
Age-group						
18–<25 *	14	4 (28.6)	Ref		Ref	
25–<45	36	11 (30.6)	1.05 (0.28–3.88)	0.891	0.50 (0.10–2.42)	0.402
45–65	11	3 (27.3)	0.96 (0.18–5.07)	0.943	0.49 (0.08–3.17)	0.380

N = number of women, CI = confidence interval, * reference strata, ** excluded during the analysis.

## Data Availability

The data presented in this study are available on request from the corresponding author due to ethical considerations.

## Supplementary Materials

The following supporting information can be downloaded at: https://www.mdpi.com/article/10.3390/tropicalmed10110317/s1. Figure S1: Frequencies of naïve, central memory, effector memory and terminally differentiated memory CD4^+^ T cells stratified by age groups between WB+ and WB− women in the peripheral blood (18–<25 WB+ *n* = 4, 18–<25 WB− *n* = 12, 25–<45 WB+ *n* = 16, 25–<45 WB− *n* = 8, 45–65 WB+ *n* = 6, 45–65 WB− *n* = 5). Groups were compared using the Mann–Whitney U-test; Figure S2: Gating strategies for peripheral blood CCR5 on total and memory CD4 T cells; Figure S3. Comparison of CD4 T cells expressing CCR5 categorised by age. Frequencies of CD4 T cells expressing CCR5 (A), memory CD4 T cells expressing CCR5 (B) and activated memory CD4 T cells expressing CCR5 (C) between WB+ and WB− among three age groups: 18–<25 (*n* = 3 WB+, 7 WB−), 25–<45 (*n* = 15 WB+, 8 WB−) and 45–65 (*n* = 5 WB+, 2 WB−). Groups were compared using the Mann–Whitney U-test; Figure S4. Mean Fluorescence Intensity (MFI) of CCR5+ concerning WB infection status. MFI of CCR5+ in the total CD4 T cells (A) and in the memory CD4 T cells (B). Each grey square and blue diamond represents a participant, and the horizontal line is the group median. Groups were compared using the Mann–Whitney U-test; Figure S5. Gating strategies for peripheral blood α4β7 on total, memory and activated memory CD4 T cells; Figure S6. Frequencies of α4β7^+^ CD4 T cells in cervical mucosal and peripheral blood concerning WB infection status. Representative flow plots and a graph comparing frequencies of memory CD4 T cells (A) and memory activated CD4 T cells (B) expressing α4β7 in the cervical mucosa and peripheral blood from WB+ and WB− groups. Cx: WB+ *n* = 23, Cx: WB− *n* = 17, Pb: WB+ *n* = 26, and Pb: WB− *n* = 25. Each grey square and blue diamond represents a participant, and a horizontal line is the group median. Groups were compared using the Mann–Whitney U-test; Figure S7: Gating strategies for the mucosal CD4 T cells and the two populations of γδ T cells.

## Abbreviations

The following abbreviations are used in this manuscript:

Cx	Cervical mucosa
FRT	Female reproductive tract
HIV	Human Immunodeficiency Virus
HR HPV	High-Risk Human Papillomavirus
Pb	Peripheral blood
STI	Sexually transmitted infection
WB+	*Wuchereria Bancroft*-infected
WB−	*Wuchereria bancrofti*-uninfected
WLWH	Women living with HIV
WLWoH	Women living without HIV
