# Supplementary material for: Impact of Wuchereria bancrofti Infection on Cervical Mucosal Immunity and Human Papillomavirus Prevalence in Women from Lindi and Mbeya Regions, Tanzania
Source: Trop Med Infect Dis. 2025 Nov 10;10(11):317. doi: 10.3390/tropicalmed10110317 (PMC12656326; doi:10.3390/tropicalmed10110317)
Supplement: Supplementary file 1 [file tropicalmed-10-00317-s001.zip › tropicalmed-3851780-supplementary.pptx]

## Slide 1
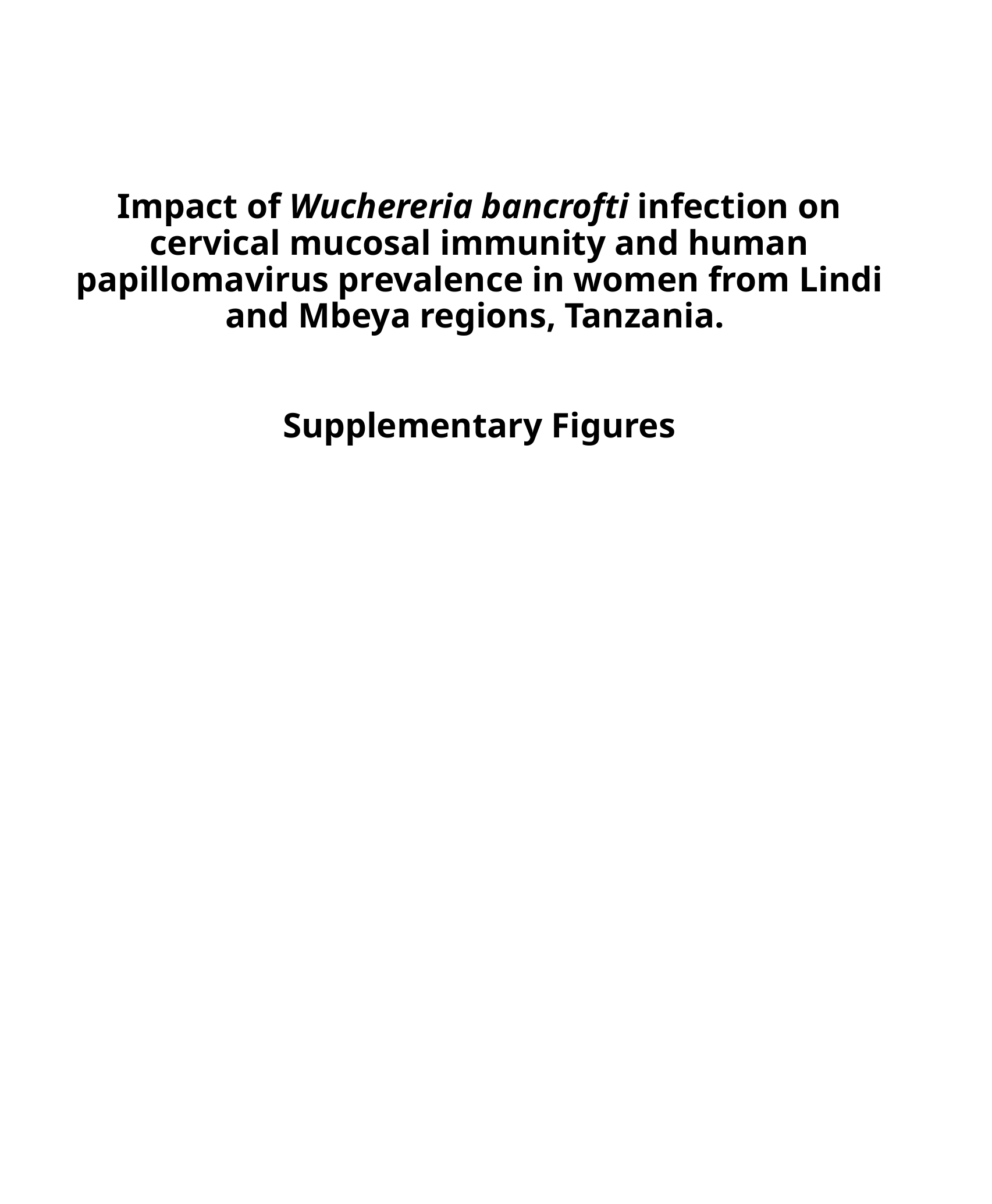

# Impact of Wuchereria bancrofti infection on cervical mucosal immunity and human papillomavirus prevalence in women from Lindi and Mbeya regions, Tanzania. Supplementary Figures

## Slide 2
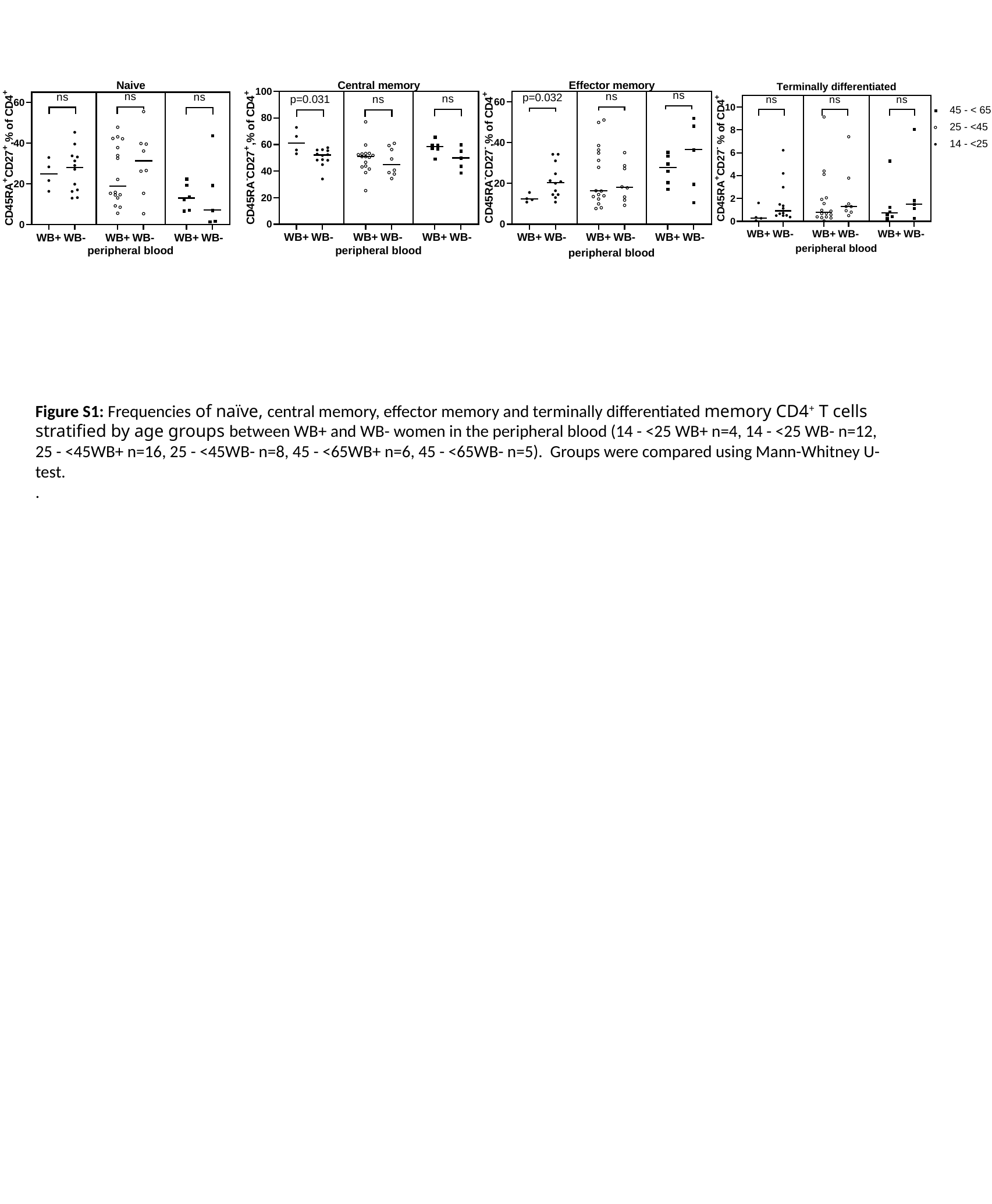

Figure S1: Frequencies of naïve, central memory, effector memory and terminally differentiated memory CD4+ T cells stratified by age groups between WB+ and WB- women in the peripheral blood (14 - <25 WB+ n=4, 14 - <25 WB- n=12, 25 - <45WB+ n=16, 25 - <45WB- n=8, 45 - <65WB+ n=6, 45 - <65WB- n=5). Groups were compared using Mann-Whitney U-test.
.

## Slide 3
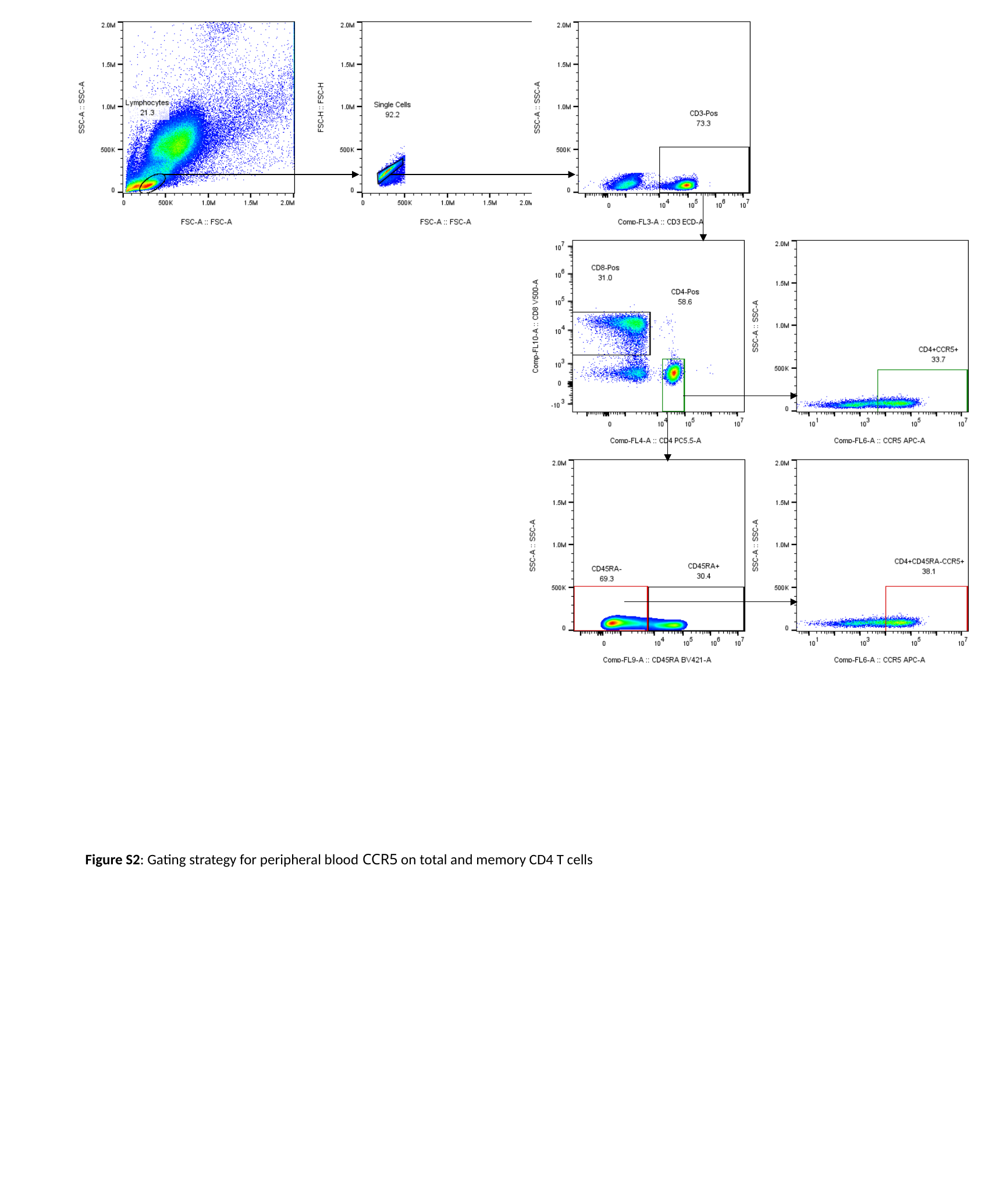

Figure S2: Gating strategy for peripheral blood CCR5 on total and memory CD4 T cells

## Slide 4
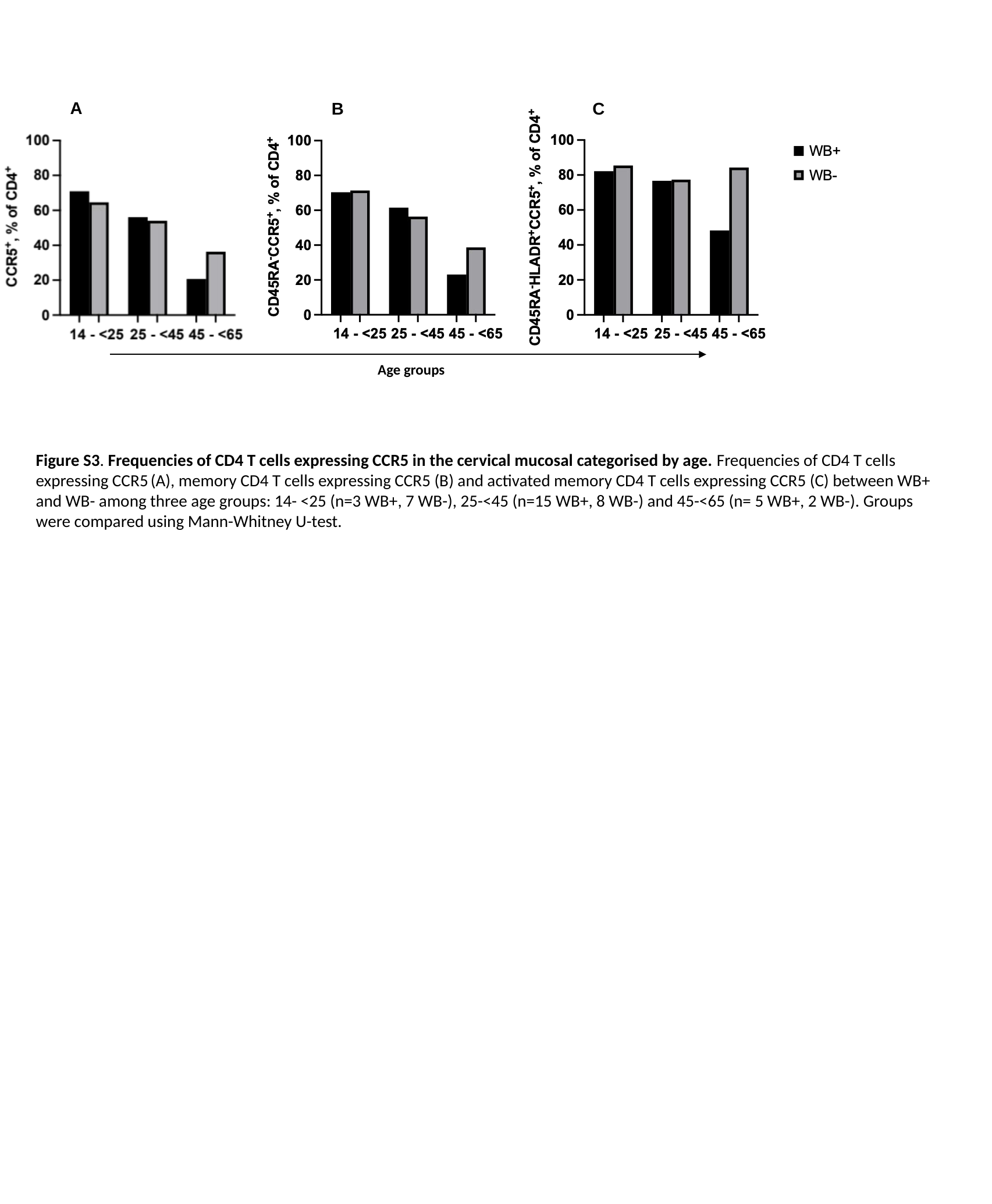

A
B
C
Age groups
Figure S3. Frequencies of CD4 T cells expressing CCR5 in the cervical mucosal categorised by age. Frequencies of CD4 T cells expressing CCR5 (A), memory CD4 T cells expressing CCR5 (B) and activated memory CD4 T cells expressing CCR5 (C) between WB+ and WB- among three age groups: 14- <25 (n=3 WB+, 7 WB-), 25-<45 (n=15 WB+, 8 WB-) and 45-<65 (n= 5 WB+, 2 WB-). Groups were compared using Mann-Whitney U-test.

## Slide 5
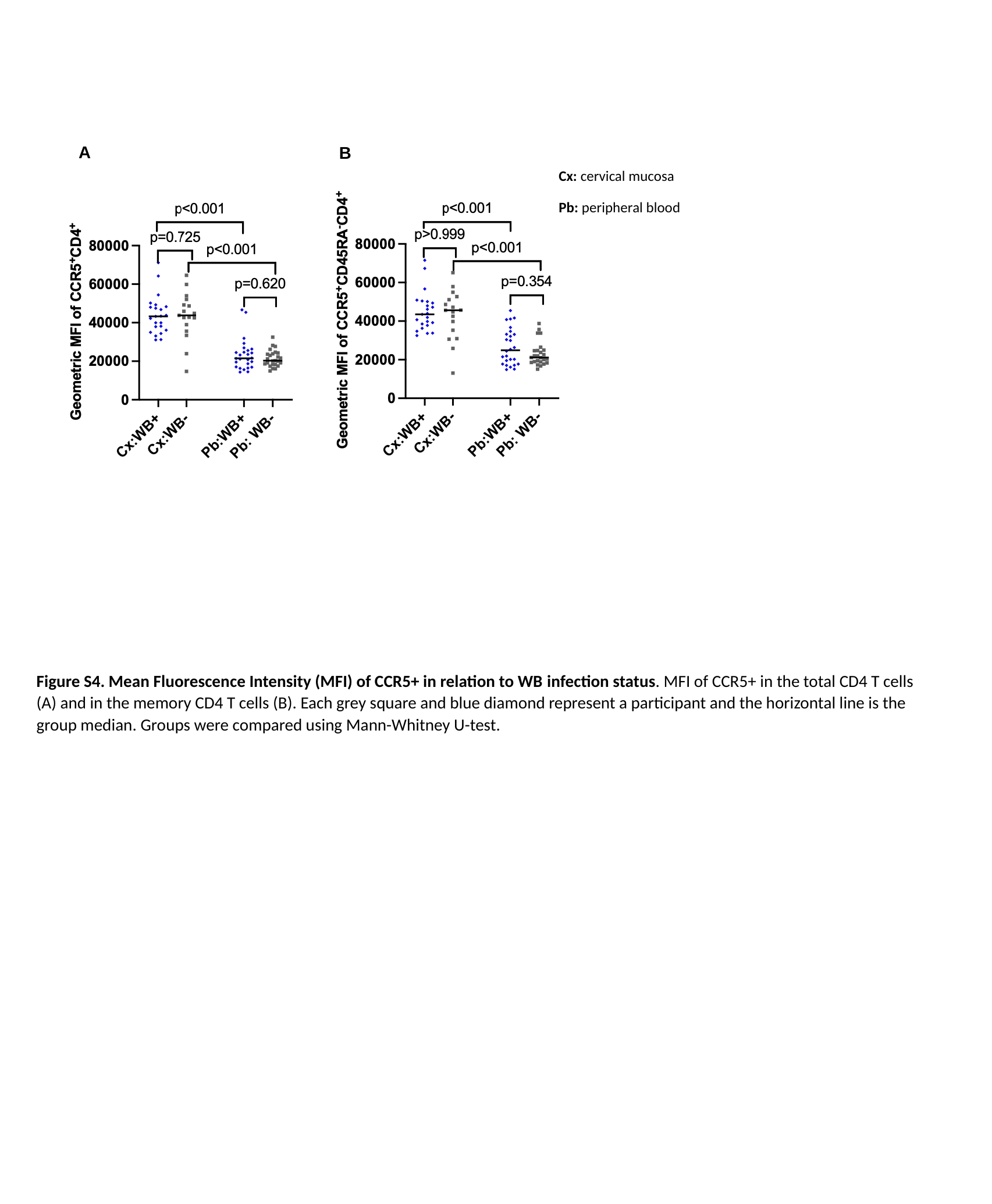

A
B
Cx: cervical mucosa
Pb: peripheral blood
Figure S4. Mean Fluorescence Intensity (MFI) of CCR5+ in relation to WB infection status. MFI of CCR5+ in the total CD4 T cells (A) and in the memory CD4 T cells (B). Each grey square and blue diamond represent a participant and the horizontal line is the group median. Groups were compared using Mann-Whitney U-test.

## Slide 6
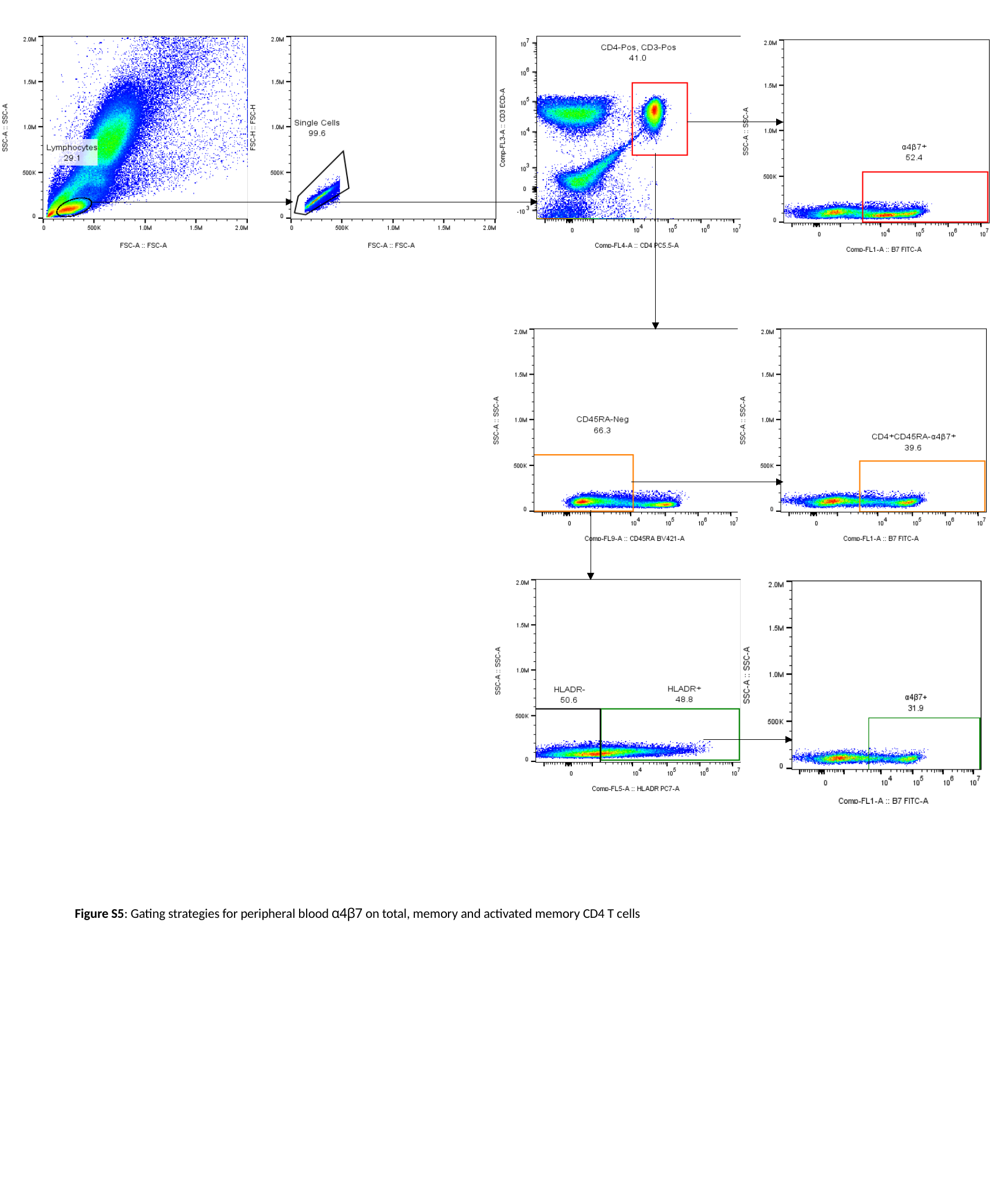

Figure S5: Gating strategies for peripheral blood α4β7 on total, memory and activated memory CD4 T cells

## Slide 7
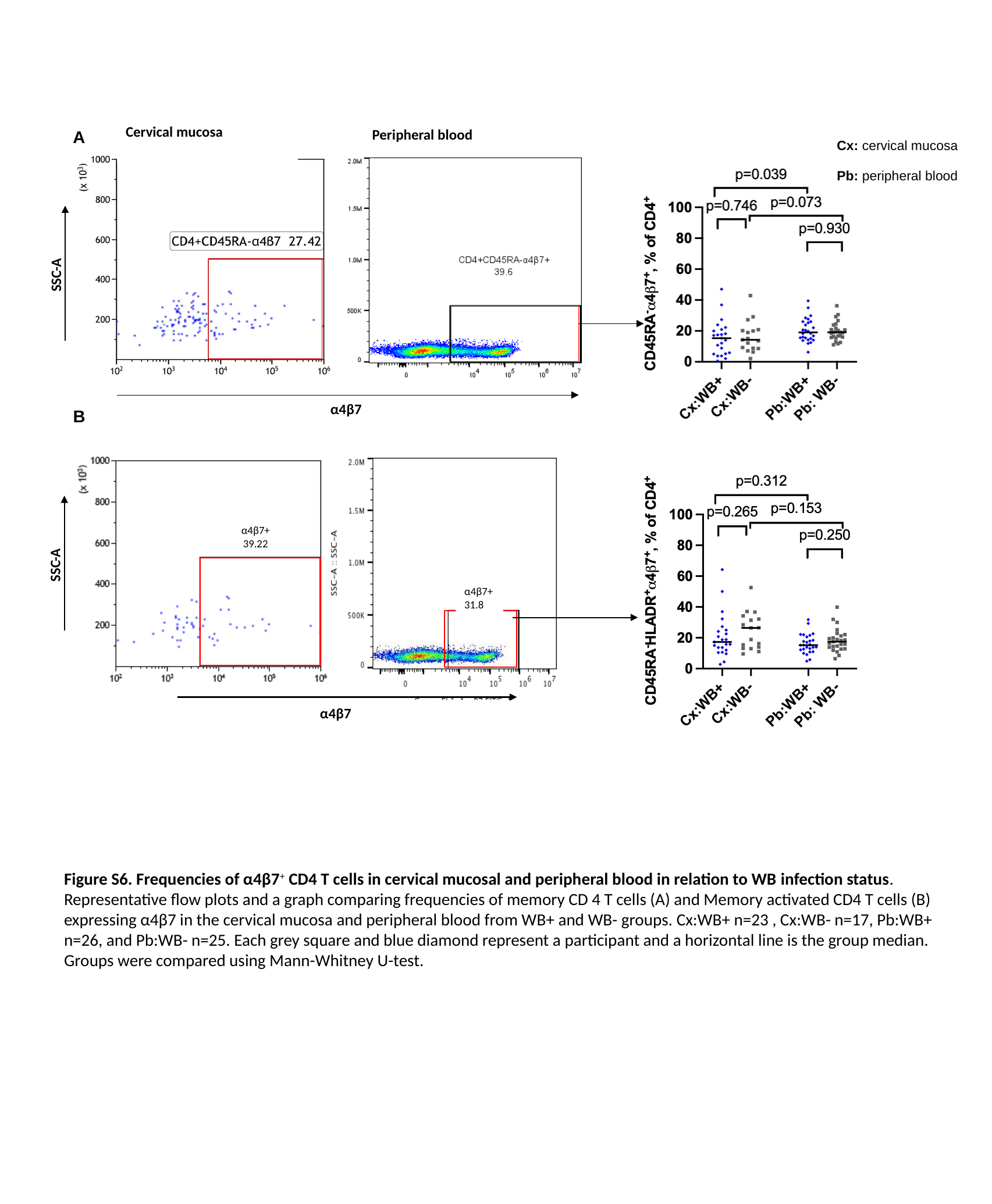

Cervical mucosa
Peripheral blood
A
Cx: cervical mucosa
Pb: peripheral blood
 SSC-A
α4β7
B
 α4β7+
31.8
 α4β7
α4β7+
39.22
α4β7+
31.8
 SSC-A
Figure S6. Frequencies of α4β7+ CD4 T cells in cervical mucosal and peripheral blood in relation to WB infection status. Representative flow plots and a graph comparing frequencies of memory CD 4 T cells (A) and Memory activated CD4 T cells (B) expressing α4β7 in the cervical mucosa and peripheral blood from WB+ and WB- groups. Cx:WB+ n=23 , Cx:WB- n=17, Pb:WB+ n=26, and Pb:WB- n=25. Each grey square and blue diamond represent a participant and a horizontal line is the group median. Groups were compared using Mann-Whitney U-test.

## Slide 8
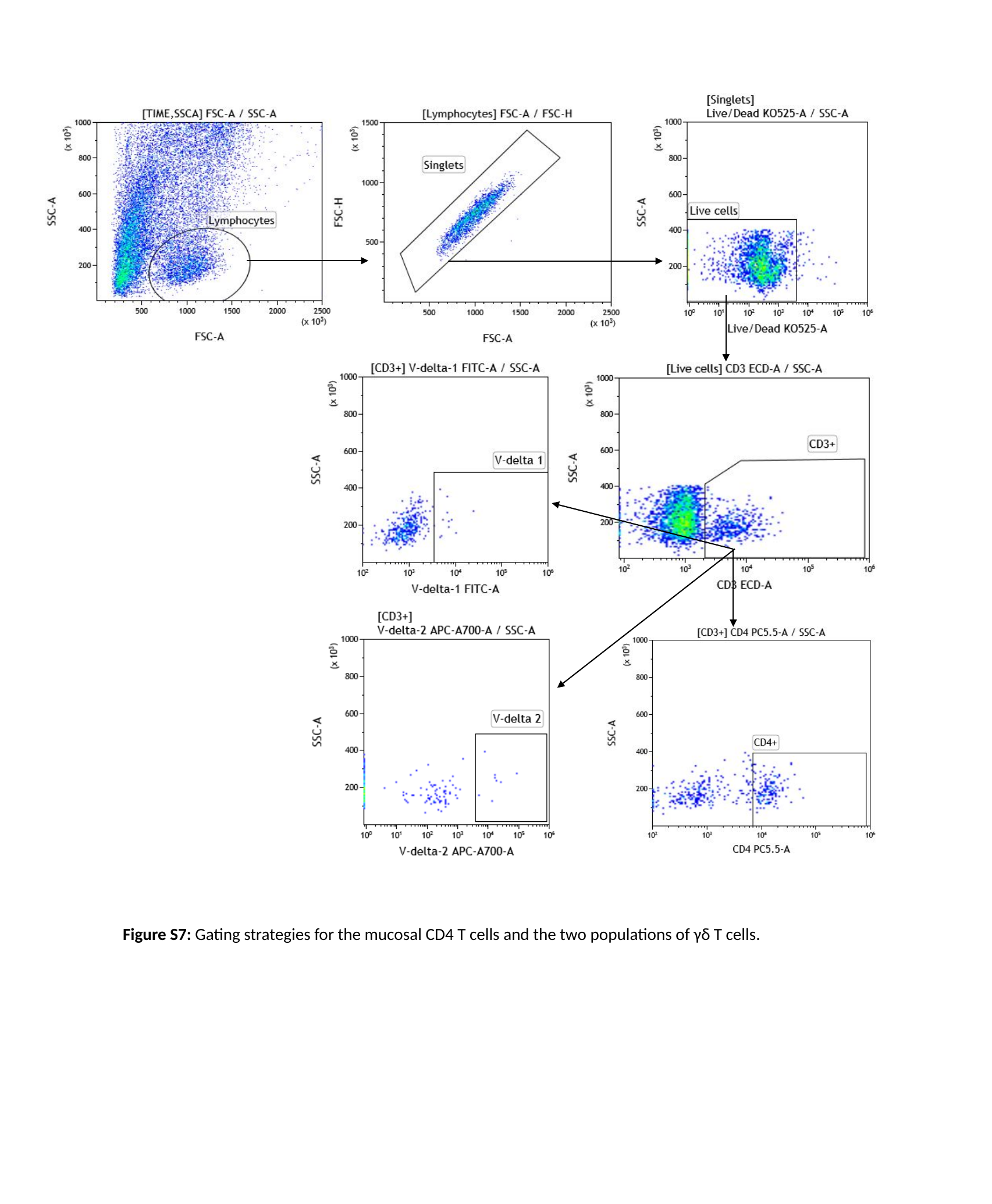

Figure S7: Gating strategies for the mucosal CD4 T cells and the two populations of γδ T cells.
